# Pseudotype Neutralization Assays: From Laboratory Bench to Data Analysis

**DOI:** 10.3390/mps1010008

**Published:** 2018-01-22

**Authors:** Francesca Ferrara, Nigel Temperton

**Affiliations:** Viral Pseudotype Unit, Medway School of Pharmacy, The Universities of Greenwich and Kent at Medway, Chatham ME4 4TB, UK; ff63@kentforlife.net

**Keywords:** influenza, pseudotypes, serology, lentiviral vector, antibodies, neutralization

## Abstract

Pseudotype neutralization assays are powerful tools to study functional antibody responses against viruses in low biosafety laboratories. However, protocols described in the literature differ widely with respect to material, reagents, and methods used to perform these assays and to analyse the raw data generated. This could result in discrepancies between the results of different laboratories even when the same pseudotypes and the same samples are analysed. Here, we describe, in detail, an experimental protocol to perform pseudotype neutralization assays using lentiviral pseudotypes bearing influenza haemagglutinin and expressing firefly luciferase. We also present the steps necessary to analyse the data and calculate the half maximal inhibitory concentration of the sera analysed. This protocol will provide support for the validation and the standardization of the pseudotype neutralization assay for influenza virus serology. Additionally, it will provide a starting point for the development of pseudotype neutralization assays using pseudotypes bearing other viral envelope proteins.

## 1. Introduction

Neutralization assays are powerful tools to study human and animal antibody responses against viruses elicited by vaccination or natural exposure [1]. These assays routinely require the use of wild-type viruses, and this limits their application when highly pathogenic human viruses are studied. However, replication-deficient viruses can often be used as a safe alternative. When enveloped viruses are studied, gammaretroviral and lentiviral vectors pseudotyped with the viral glycoproteins of interest [2] are a feasible substitute for viruses. Numerous lentiviral vectors harboring glycoproteins of different viruses have been described in the literature, and some of them have been used in neutralization assays [3,4]. For example, the use of pseudotyped lentiviral vectors as surrogate antigens in neutralization assays is extremely useful in influenza research. Influenza pseudotype neutralization assays are a safe alternative when potentially pandemic viruses need to be studied [5]. Furthermore, they have been shown to have a high sensitivity in detecting antibodies directed against the stalk of influenza envelope glycoprotein, the haemmaglutinin [6]. These antibodies are the ones that new influenza universal vaccination candidates are intended to elicit [7]. Extended information on influenza pseudotype neutralization assay variables has recently been systematically reviewed [8]. However, a standard operating procedure-like protocol is not yet available in the literature, limiting the application and standardization of this assay. Here we present a detailed protocol to perform pseudotype neutralization assays, using influenza haemagglutinin-enveloped HIV pseudotypes carrying a firefly luciferase reporter. The use of such a reporter results in a wide dynamic range of neutralization titres and a high level of sensitivity. The alternative green fluorescent protein (GFP) reporter would necessitate either a 96-well flow cytometer, or the counting of green “transduced” cells with a fluorescence microscope. This protocol was designed to be easily adapted to other lentiviral pseudotype platforms, and will provide support for the validation and the standardization of the pseudotype neutralization assay for influenza virus serology. The data from this assay can be harmonized with that obtained by other neutralization assays, by making use of common reference reagents, such as those available from the National Institute for Biological Standards and Control (NIBSC) and the World Organisation for Animal Health (OiE). Additionally, this protocol will provide a starting point for the development of pseudotype neutralization assays using lentiviral pseudotypes bearing other viral envelope proteins.

## 2. Experimental Design

### 2.1. General Considerations

The pseudotype neutralization assay, here described, is a cell-based viral neutralization assay that is performed in a 96-well format. 

**Figure 1:**
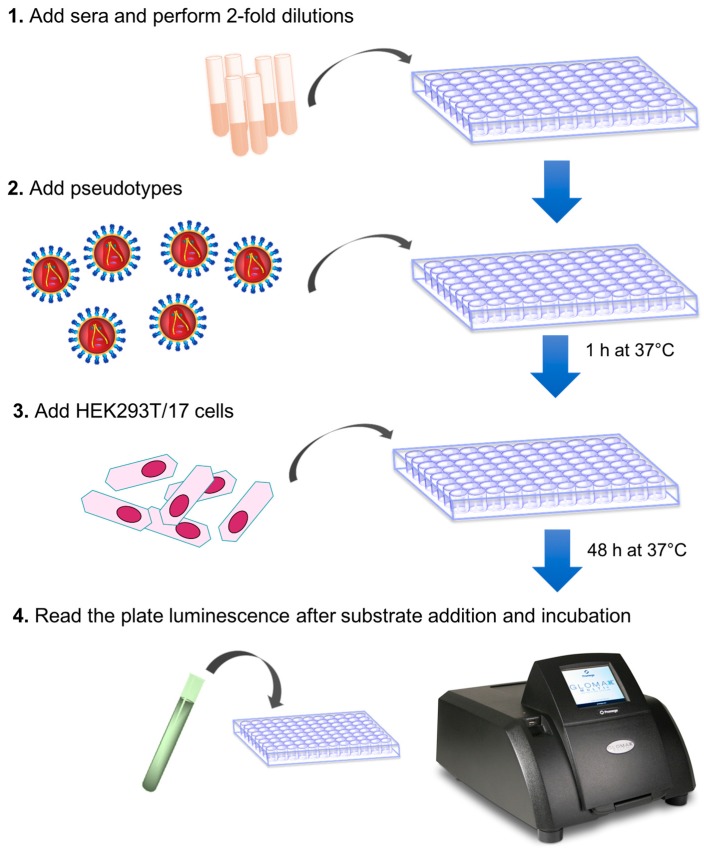
Schematic representation of the pseudotype neutralization assay.

Figure 1 is a schematic representation of the main assay steps. Similar to a traditional microneutralization assay, different concentrations of antibodies or sera are incubated with cells and pseudotype particles. The pseudotype lentiviral particles used for this assay encode firefly luciferase in their lentiviral vector genome. When their genome integrates after entry into cells, firefly luciferase expression and activity is proportional to the number of cells that were transduced.

After 48 h incubation, to permit cell transduction of non-neutralized particles and expression of firefly luciferase, cells are lysed in the presence of a luciferase substrate to assess luciferase activity. The comparison between the luciferase signals detected in untransduced cells, in cells transduced with pseudotypes only, and in cells transduced with pseudotypes in the presence of serum, will enable one to determine if that serum has neutralization activity against the pseudotype tested.

### 2.2. Materials

- 12-channel micro-pipette
- 8-channel micro-pipette
- Pipettes (10–200 µL)
- 200 µL MultiGuard NX Barrier tips (Sorenson BioScience, Salt Lake City, UT, USA; Cat. no.: 30550T)
- 20 µL MultiGuard Barrier tips (Sorenson BioScience; Cat. no. 35220)
- 10 µL MultiGuard E Barrier tips (Sorenson BioScience; Cat. no. 15020T)
- Dulbecco’s Modified Eagle Medium (DMEM) with high glucose and GlutaMAX (Thermo Fisher Scientific Inc., Waltham, MA, USA; Cat. no.: 31966-021; or Sigma-Aldrich, St. Louis, MO, USA; Cat. no.: D6429; or PAN Biotech, Aidenbach, Bavaria, Germany; Cat. no.: P04 04510)
- Fetal Bovine Serum (FBS; Thermo Fisher Scientific Inc.; Cat. no.: 10500-064; or PAN Biotech; Cat. no.: P30 8500)
- Penicillin/streptomycin (Sigma-Aldrich; Cat. No: P4333).
- 0.05% (*w/v*) Trypsin 0.53 mM EDTA solution (Sigma-Aldrich; Cat. no.: T3924; or PAN Biotech; Cat. no.: P10 040100)
- Nunc F96 MicroWell white polystyrene plates (Thermo Fisher Scientific Inc.; Cat. no.: 136101)
- Reagent reservoirs (Corning Inc., Corning, NY, USA; Cat. no.: 4870; or Dutscher Scientific, Brumath, France; Cat. no.: 006793)
- Influenza pseudotyped lentiviral vectors encoding Firefly luciferase with known titer expressed as Relative Luminescence Units (RLU)/mL [8,9,10]
- Positive control sera (appropriate for the pseudotyped lentiviral vectors used, see Section 2.6)
- Negative control sera (appropriate for the pseudotyped lentiviral vectors used, see Section 2.6)
- Bright Glo Luciferase Assay System (Promega, Madison, WI, USA; Cat. no.: E2650)

### 2.3. Cell Line

For influenza pseudotype neutralization, we used HEK293T/17 cells (ATCC, CRL­11268) as target cell lines [9]. If necessary, the assay can be modified to allow the use of a different cell line (MDCK for example); in such cases, the lentiviral vector input and number of cells used should be optimized.

HEK293T/17 cells used for the assay should be cultured and maintained in DMEM with high glucose and GlutaMAX and with 15% (*v/v*) FBS and 1% (*v/v*) penicillin/streptomycin. We recommend performing HEK293T/17 cell subculture, keeping the ratios and days of subculture constant. In general, cells can be subcultured, depending on cell growth of the particular batch and the vessel used, on Monday and Wednesday, at ratios of 1:4 or 1:5, and on Friday, at ratios of 1:8 or 1:10. If cells are thawed gently and a good recovery and viability is observed after thawing, the assay can be performed with cells from passage 2. Cells are usually kept in culture and used for 3–4 months (equivalent to passage 36–40). In any case, if changes in cell morphology or growth are observed, the cell batch should be discarded, and a new vial should be thawed. 

In contrast to the HEK293T/17 cell culture medium, the complete medium used in the assay itself is prepared by adding FBS at a final concentration of 10% (*v/v*) and 1% (*v/v*) penicillin/streptomycin to DMEM with high glucose and GlutaMAX. This is done to avoid non-specific neutralization by FBS. If non-specific neutralization is observed often, it is advisable to use FBS at a concentration of 5% (*v/v*).

### 2.4. Equipment and Software

- GloMax Multi detection system luminometer (Promega; Cat. no.: E7031 and E7041) or equivalent
- Centrifuge for 96-well plates
- Humidified CO_2_ Incubator
- Biological Safety Cabinet
- Automated cell counter (Bio-Rad Laboratories Inc., Hercules, CA, USA; Cat. no.: 1450102) or cell counting chamber slides
- VACUSIP (INTEGRA Biosciences, Hudson, NH, USA; Cat. no.: 159000) or vacuum pump or similar instrument (optional)
- GraphPad Prism version 6 Software (GraphPad Software Inc., La Jolla, CA, USA)

### 2.5. Plate Format

**Figure 2:**
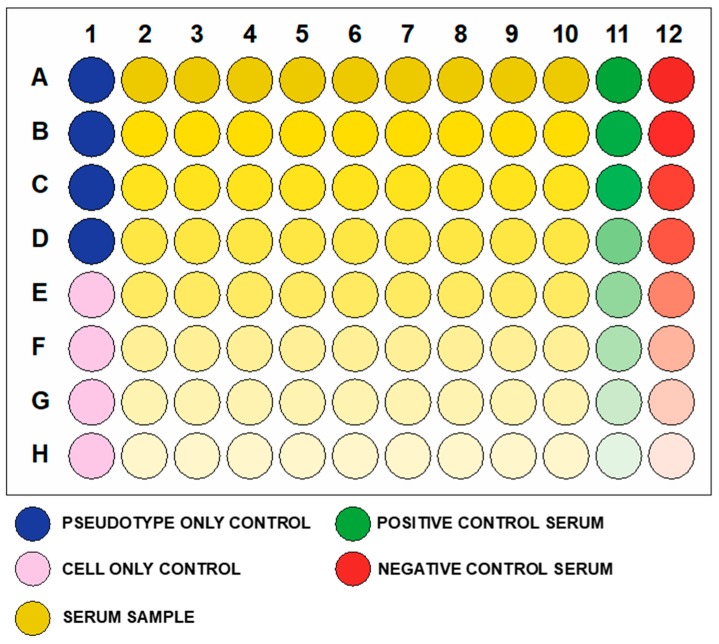
Pseudotype neutralization assay 96-well plate format. Colour gradients represent dilution of the serum samples down the plate. Usually, a 1:2 serial dilution starting with 1:40 or 1:80 is used.

In Figure 2, we report the plate format that is used in the pseudotype neutralization protocol described. 

A serial dilution of 1:2 of an appropriate positive control (serum or antibodies) that can neutralize the pseudotype that will be used in the assay is included in each plate, to check that the pseudotype used is correctly neutralized. Isotype control antibodies or serum are also included and diluted, to verify that non-specific neutralization/non-specific background signal is not detected.

In this protocol, we will show the calculation for one plate. If more than nine samples need to be analyzed and multiple plates are necessary, multiply the calculation here reported, using a factor corresponding to the number of plates required.

To maintain adequate reagent volumes and make pipetting more straightforward, we recommend doing all the calculations with an assumption of 100 wells for each plate.

### 2.6. Sera

Common, commercially available sera, neutralizing the influenza virus of the strain and subtype of interest, can be used as positive controls in a research-based setting. For negative controls, commercially available serum of naïve animals or isotype controls that does not possess influenza virus neutralization activity can be used. If using commercially available monoclonal antibodies that can neutralize multiple strains of influenza virus (e.g., C179 Takara Bio USA Inc., Mountain View, CA, USA; Cat. no.: M145), it should be kept in consideration that their half maximal inhibitory concentration (IC_50_) in this assay is lower than in classic neutralization assays.

Human sera, or sera from different animal origins can be used in this assay. Sera treatment, such as heat inactivation, can be performed and usually does not affect the assay results. Other treatments, such as receptor destroying enzymes, were not tested. 

## 3. Procedure

### 3.1. Assay Set-Up. Time for Completion: 2:30 h

1. Before performing the assay, calculate the amount of influenza pseudotyped lentiviral vector that will be used.
  - Firstly, calculate how many RLU are required for one assay, performed in one plate. In each well, we recommend a virus input that results in 1 × 10^6^ RLU:
    $$1\times 10^{6}\;RLU\times 100\;wells=1\times 10^{8}\;RLU$$
  - Therefore, the volume of pseudotype (*P*) that should be used is calculated, as shown below:
    $$P=\frac{1\times 10^{8}\;RLU}{pseudotype\;titre\;\left(RLU/\mathrm{mL}\right)}$$
  - Considering that pseudotypes are added to each well at a volume of 50 µL for one plate, 5 mL (50 µL × 100) of pseudotype suspension should be prepared by adding to the volume calculated in b., bringing the complete medium to a total volume of 5 mL.
2. Add 50 µL of complete medium to A1, B1, C1, D1.
3. Add 100 µL to E1, F1, G1, H1 (Cell only control).
4. Add 50 µL of medium to B2-H12 (A2–A12 should remain empty in this step).
5. Add 5 µL (or 2.5 µL or 2 µL or less) of each serum sample to wells A2 to A10.
  Note: if the same serum will be analyzed multiple times to obtain technical replicates, we advise putting the replicates in adjacent columns to simplify the subsequent analysis.
6. Add to A11, the same amount (5 µL or 2.5 µL or 2 µL or less) of positive control serum and to A12, the same amount of negative control serum.
7. Make volume up to 100 µL with the complete medium in A2–A12.
8. **OPTIONAL STEP** Spin the plate in a centrifuge for 1 min at 500× *g*.
9. Take 50 µL from wells A2–A12 (samples and serum control) and serially dilute down the plate (from A to H) using a 12-channel pipettor.
10. Spin the plate in a centrifuge for 1 min at 500× *g*.
11. In a new reservoir, prepare the pseudotyped lentiviral vector on the basis of the calculation performed in step 1: add the pseudotype volume and then the complete medium, to give a final volume of 5 mL. Mix by gently moving the reservoir and by pipetting up and down with the multichannel pipette.
12. Add 50 µL of the pseudotyped lentiviral vector to each well, with the exception of E1, F1, G1, H1 (cell only control).
  A1, B1, C1 and D1 will be the pseudotype only (no sera) control.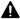 **CRITICAL STEP** To avoid carry over, change tips for each column or row of the plates when adding the pseudotyped lentiviral vector.
13. Spin the plate in a centrifuge for 1 min at 500× *g*.
  NOTE: Manual mixing is not required after this step.
14. Incubate the serum-pseudotype mix for 1 h at 37 °C and 5% CO_2._
15. Fifteen minutes before the end of the 1-hour incubation time, prepare a HEK293T/17 cell suspension (assay cell suspension) directly in a sterile reservoir.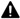 **CRITICAL STEP** Do not use tubes to prepare the cell suspension as HEK293T/17 can tend to clump, and this could result in a non-homogenous cell suspension. Keep cells in the tissue culture dish during the preparation of the cell suspension.
  - Remove media from a 95–100% confluent HEK293T/17 cell dish.
  - To wash the cells and neutralize remaining serum, add 2 mL of Trypsin-EDTA, and then remove it.
  - Add 2 mL of Trypsin-EDTA.
  - Incubate the dish for 5 min at 37 °C and 5% CO_2._
    Neutralize the Trypsin-EDTA by adding 6 mL of complete medium.
  - Resuspend carefully, until a single cell suspension is obtained (this can require 2–3 min).
  - Count the HEK293T/17 cells, using an appropriate cell counting chamber slide under the microscope, or a cell counter, following the manufacturer’s instructions. A concentration, expressed as cells/mL, should be obtained.
  - Calculate the cells needed for one assay plate:
    $$1.5\times 10^{4}\;cells\times 100\;wells=1.5\;\times 10^{6}\;cells$$
  - Calculate the volume of cell suspension for one assay plate:
    50 μL×100 wells=5000 μL=5 mL
  - Calculate the volume (x) of HEK293T/17 cells for assay cell suspension:
    $$x=\frac{1.5\times 10^{6}\;cells}{HEK293T/17\;cell\;concentration\;\left(cells/\mathrm{mL}\right)}$$
  - Resuspend the HEK293T/17 cells in the dish and then add the volume of cells calculated (*x*) to a clean and sterile reservoir.
  - Add the volume of complete medium needed to reach a total volume of 5 mL.
  - Mix by moving the reservoir back and forward.
16. After careful mixing of the assay cell suspension by pipetting up and down, add 50 µL of HEK293T/17 cell suspension to each well of the assay plate that was removed from the incubator.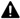 **CRITICAL STEP** To avoid carry over, change tips for each column or row of the plates when adding the cells.
17. Spin the plate in a centrifuge for 1 min at 500× *g*.
18. Incubate plate for 48 h at 37 °C and 5% CO_2._

### 3.2. Firefly Luciferase Read-Out. Time for Completion: 15 min

19. Assay firefly luciferase RLU, using a Promega GloMax Multi detection system luminometer or equivalent.
  - Remove plate from the incubator.
  - Add 50 µL of Bright-Glo, reconstituted following the manufacturer’s instructions, to each well of the 96-well plate.
  - Incubate for 5 min at room temperature.
  - Read, using the Bright-Glo protocol, pre-installed on the GloMax Multi detection system luminometer (or alternative machine, using readily available settings for firefly luciferase).
  - Export the data onto a USB stick for further analysis on a PC.
  OR
  f. Remove plate from the incubator.
  g. Remove the medium from each well of the plate (vacuum pump or similar instrument).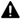 **CRITICAL STEP** To avoid carry over, change the tips for each column or row of the plates when removing the media.
  h. Add 50 µL of a reading solution, previously prepared by a 1:2 dilution of Bright Glo in complete medium (i.e., 2.5 mL of Bright Glo and 2.5 mL of complete medium).
  i. Incubate for 5 min at room temperature.
  j. Read using the Bright-Glo protocol pre-installed on the GloMax Multi detection system luminometer (or alternative machine, using readily available settings for firefly luciferase)
  k. Export the data onto a USB stick for further analysis on a PC.

### 3.3. Data Analysis. Time for Completion: 01:30 h

Microsoft Excel (Microsoft Corporation, Redmond, WA, USA) and GraphPad Prism should be used to analyse the data and calculate the half maximal inhibitory dilution (ID50) of each serum tested, or the half maximal inhibitory concentration (IC50) of antibodies tested. Microsoft Excel is necessary to perform some of the calculations, and to visualize the raw data, as the software can automatically open the comma-separated values (csv) file that is generated by the GloMax Multi detection system luminometer. The protocol steps reported here for the analysis are performed using GraphPad Prism version 6. Differences in the procedure may be necessary if other versions of the software are used.

**Figure 3:**
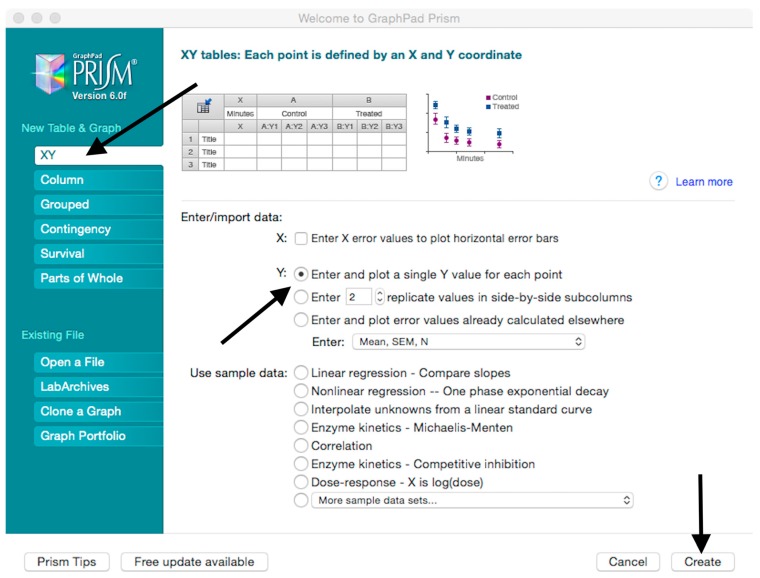
Instructions for creating a new GraphPad Prism (GraphPad Software Inc., La Jolla, CA, USA) file to be used for data analysis.

**Figure 4:**
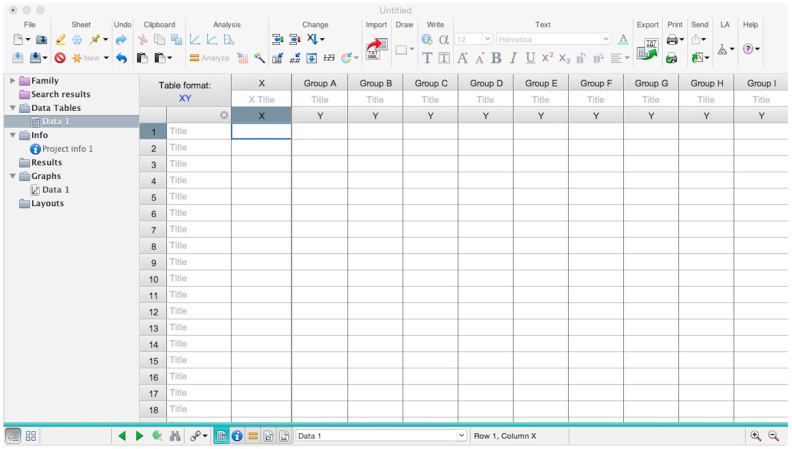
Example of GraphPad Prism table that is used to input and analyse the data.

**Figure 5:**
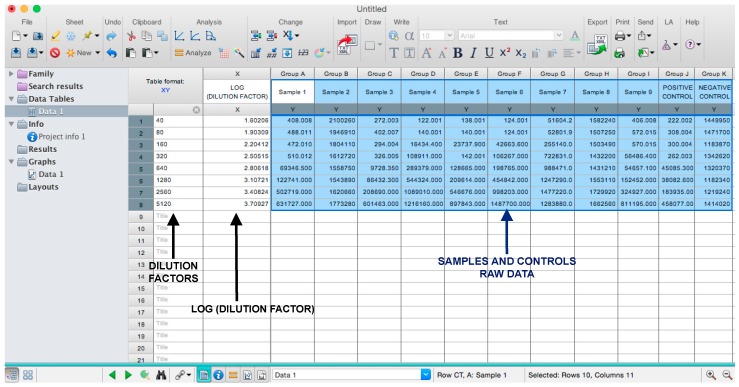
Example of a GraphPad Prism table in which the raw data and serum dilution factors were appended to perform the data analysis.

**Figure 6:**
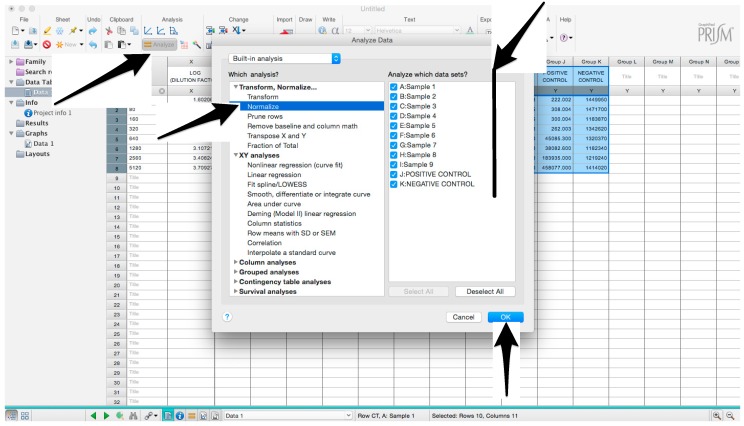
Graphical instruction for permitting the normalization of the raw data.

**Figure 7:**
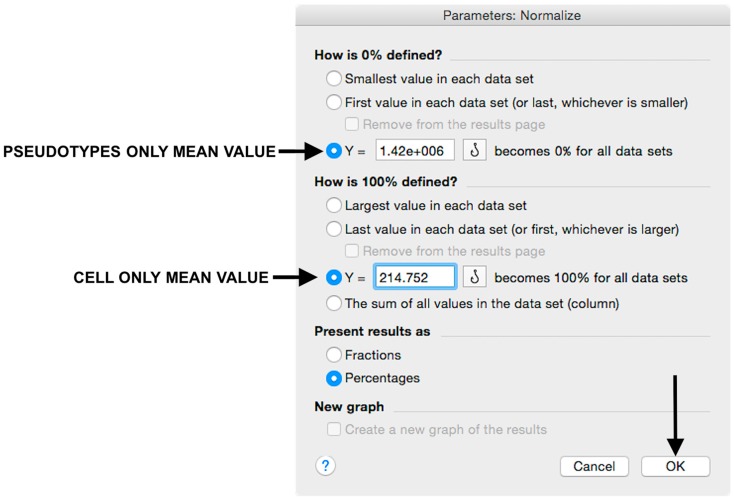
Raw data normalization using “pseudotype only” and “cell only” mean values in the appropriate fields.

**Figure 8:**
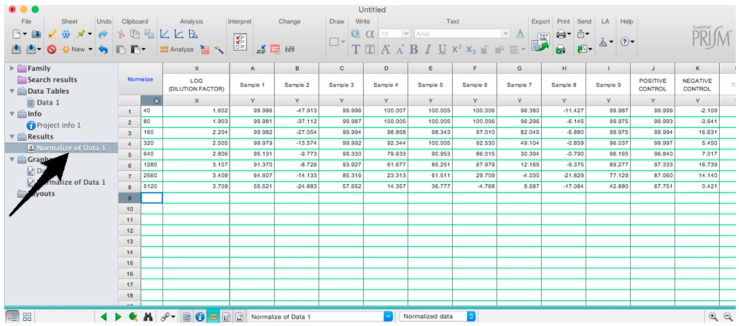
Selection of the Normalize data table.

**Figure 9:**
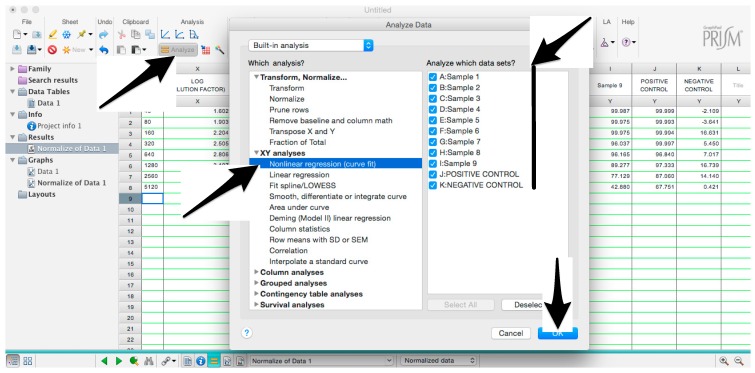
Analysis of the normalized raw data to calculate the half maximal inhibitory concentration (IC_50_) of each sera.

**Figure 10:**
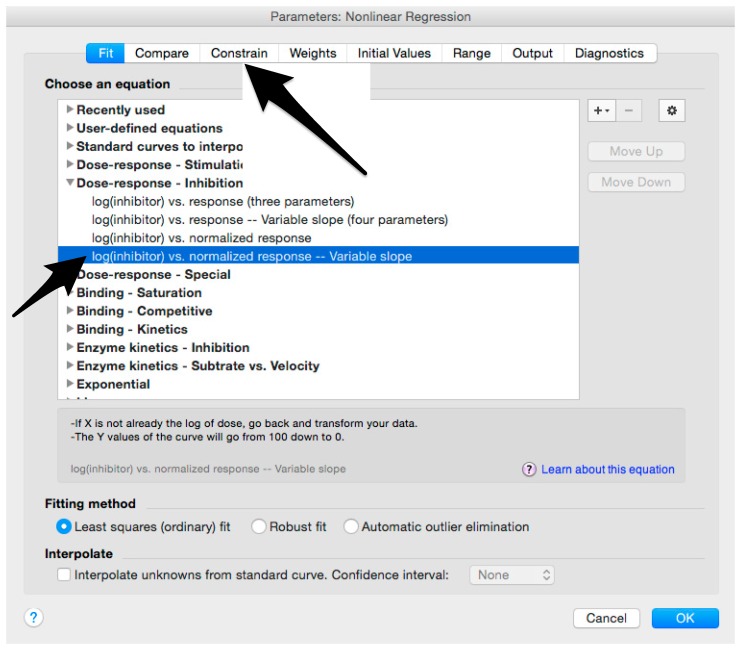
Selection of the appropriate nonlinear regression equation used to fit the data.

**Figure 11:**
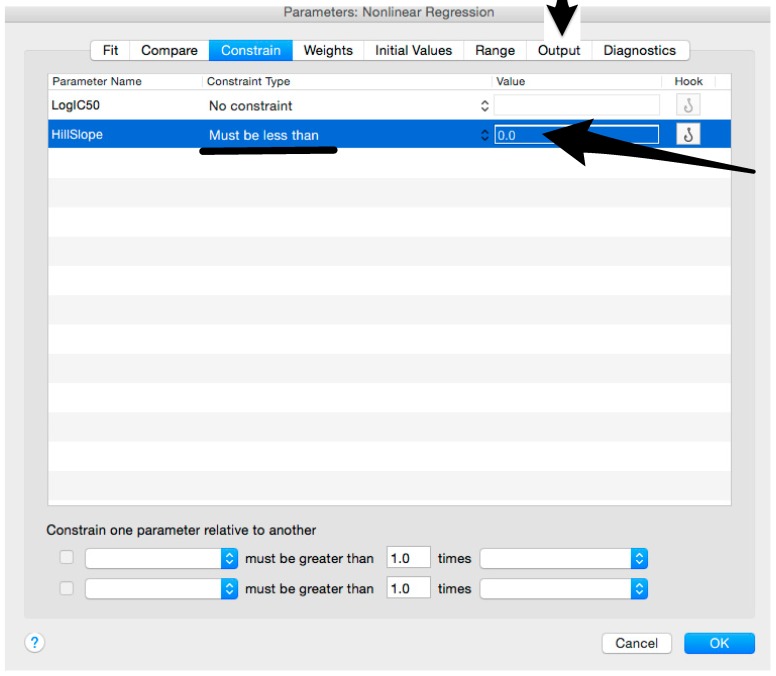
Constraints of the Hill Slope value.

**Figure 12:**
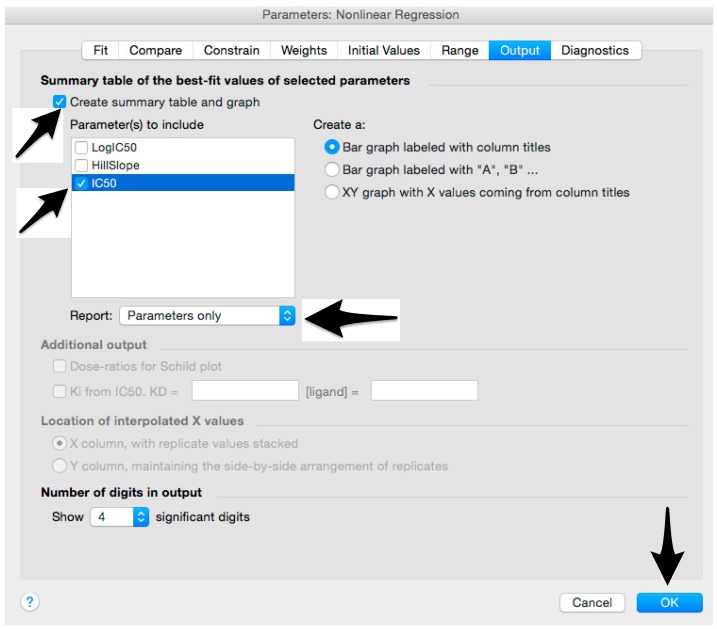
Generation of the summary table that will report the IC_50_ values.

**Figure 13:**
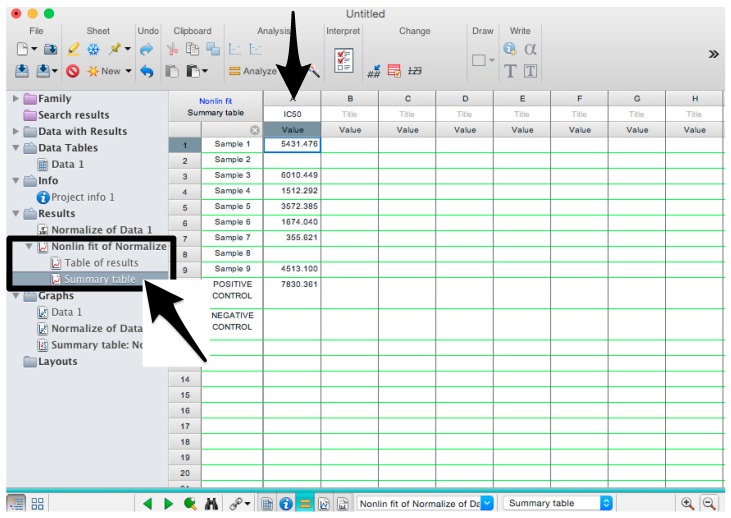
Summary table with IC_50_ values.

20. Open the raw data file using Microsoft Excel and calculate the average values of the pseudotype only control wells and of the cell only wells.
21. Open GraphPad Prism version 6.
22. Create a New project file selecting “XY” table and “Enter and plot a single value Y for each point” and then press the “Create button” (Figure 3).
  If multiple replicates for the same serum were performed on the same plate, select “Enter in … replicate values side-by-side subcolumns” and in the box, insert how many replicates were performed. Be careful to put replicates next to each other when the data are copied.
  If replicates were performed on different plates, we advise processing each replicate with “Enter in side-by-side subcolumns”, and then average the ID_50_/IC_50_ obtained.
23. A window like the one reported in Figure 4 will appear.
24. Insert assay dilution factors in the first column (40–5120, for example, if the serum sample volume was 5 µL).
25. Insert logarithmic dilution factors (which can be calculated using Microsoft Excel using the LOG10 function) in the X column.
26. Copy the samples, positive and negative serum control raw data (with the exception of pseudotype and cell only values) into the other columns; each column should represent a sample/control (Figure 5).
27. To transform the data into percentage neutralization, click on “Analyze”.
28. In the new window that opens, select “Normalize” and be sure that all the data sets are ticked; then click “OK” (Figure 6).
29. Insert the pseudotype only mean value (which was calculated in step 20) in “How is 0% defined?” and cell-only mean value in “How is 100% defined?”. Be sure that the “Y=” and the “Percentages” options are selected. Then click “OK” (Figure 7).
30. “Normalize of Data 1” table will be created. From now on, this table will be used for analysis (Figure 8).
31. Now click again on “Analyze”.
32. Select “Nonlinear regression (curve fit)” and be sure that all the data sets are ticked; then click “OK” (Figure 9).
33. Select “log(inhibitor) vs. normalized response—Variable slope” from “Dose-response-Inhibition”; then click on “Constrain” (Figure 10).
  NOTE: If ID20 (or IC20) or ID80 (or IC80) need to be reported, create and use a user-defined equation based on “Dose-response—Special: log(agonist) vs. response—FindECanything” (see Figure 10); after double-clicking, press “clone this equation”, change the name and edit the formula by putting “Bottom” as 0 and “Top” as 100. In “Rules for Initial Values”, be sure that the variables are as follows: “logECF” should be “*(Value of X at YMID)”, “HillSlope” should be “SIGN(YATXMAX- YATXMIN)” and F should be set as “(Initial Value, to be fit)”
  In “Default Constraints”, select “Must be less than” 0 for the “Hill-Slope” (see also step 34). Constrain the F parameter to 20 if IC20/ID20 needs to be calculated, or to 80 for the IC80/ID80 calculation, or any other number between 0 and 100.
  In “Transform to Report” change “ECF” to “ICF”.
34. Constrain the “Hill Slope” to be less than 0, as shown below; then click on “Output” (Figure 11).
  NOTE 1: Constraining the hill slope usually improves the data fit, forcing the software to consider the data points as part of inhibitory curves. However, if done improperly, it can block the IC_50_ calculation.
  NOTE 2: It is possible to use a pre-modified “log(inhibitor) vs. normalized response—Variable slope” function, in which the hill slope is already constrained. Please see Prism Manual Online to learn how to modify an already existing equation (or see step 33 NOTE as example).
35. Tick “Create summary table and graph”, and then remove all the ticks apart from “IC50” (Figure 12).
36. Select “Parameter only” and then click on “OK” (Figure 12).
37. Analyses will be performed, and new sheets will appear in results.
38. Check the “Summary table” for IC_50_ values (Figure 13).
39. Save the file.
  NOTE: If additional plates from the same experiment need to be analysed, the same file could be used; a new table should be added, and then proceed with analysis from point 3.

We advise always double-checking the data, the results, and especially the final graph generated by GraphPad Prism, to identify possible incongruences. This procedure should be considered a simplified guide for the data analysis and should not substitute a critical interpretation of the generated neutralization data.

## 4. Expected Results

**Figure 14:**
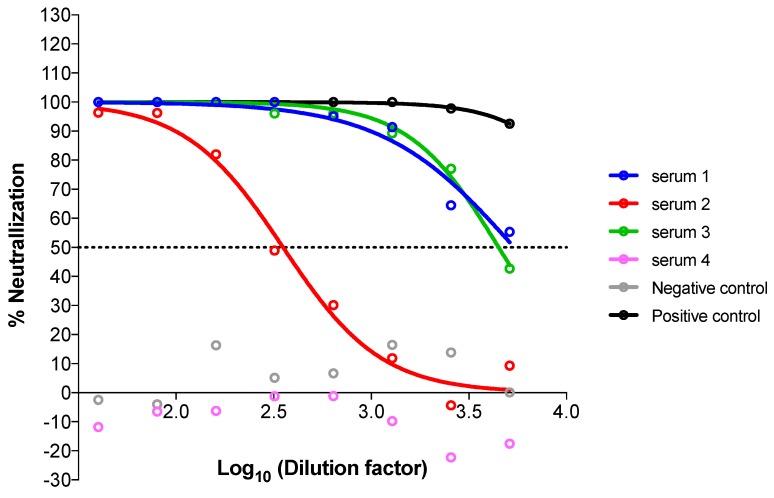
Graphical example of neutralization results of four sera after GraphPad Prism 6 analysis. Normalised percentage neutralization values are plotted against the logarithm of the dilution factors, and neutralization curves are inferred by the software that additionally calculates the IC_50_.

Figure 14 shows an example of analyzed pseudotype neutralization results. In this case, four sera were tested together with appropriate negative and positive neutralization controls. In this example, sera 1, 2 and 3 (IC_50_ serum 1 = 5402, IC_50_ serum 2 = 353, IC_50_ serum 3 = 4468) are clearly able to neutralize the pseudotype tested with different potencies, whereas serum 4 does not neutralize the pseudotype with a result comparable to the negative control.

The method with GraphPad Prism does not require having points below the 50% mark, because data are firstly normalized and then the equation is constrained to always start with 100% and always finish with 0%. Sometimes GraphPad Prism is unable to calculate the ID_50_/IC_50_, because all the points are on one of the two plateaus (for example serum 4 and negative control) and a clear curve slope cannot be computed. In that case, it does not provide a value, and the ID_50_/IC_50_ should be provided as lower or higher than the range tested.

The pseudotype neutralization assay is more sensitive than the traditional microneutralization assay—if the same serum is analyzed with a microneutralization assay, it will have lower ID_50_ values [6,9].

## 5. Conclusions and Future Work

In this paper, we present a detailed influenza pseudotype neutralization assay and downstream data analysis protocols within a unified framework. This is accompanied by extensive graphical instructions for the enumeration of neutralization titres, something that was hitherto lacking in the influenza pseudotype literature. This protocol outlines the first steps towards influenza neutralization assay standardization. It should be noted that there are additional key parameters that must be extensively evaluated to optimize an assay of this type, if validation of the assay is the end goal. These parameters are precision, accuracy, specificity, limits of detection, range, linearity and robustness. These have been addressed with an HIV-1 Env pseudotype luciferase-based neutralization assay [11], and this can provide a path forward for equivalent influenza assays. Using this protocol in a study encompassing multiple stakeholder laboratories would enable inter-laboratory variation to be studied at the parameter level.

The protocol presented can be used as an alternative or adjunct to plaque-reduction neutralization assays/microneutralization assays. It benefits from the fact that no wild-type virus is required, and therefore, containment facilities are superfluous. This is of major benefit when working with HPAI H5 and H7 viruses. It is a functional assay which measures the total neutralizing antibody response to the HA (both head and stalk). If accurate quantification of the stalk response is required, the assay can be performed using a pseudotype carrying an HA with a mismatched head (as we have described recently for H11 head/pH1 stalk pseudotypes [12]).
